# Need to teach family medicine concepts even before establishing such practice in a country

**DOI:** 10.1186/1447-056X-13-1

**Published:** 2014-01-08

**Authors:** Rasnayaka M Mudiyanse

**Affiliations:** 1 Department of Pediatrics, Faculty of Medicine University of Peradeniya, Peradeniya, Sri Lanka

**Keywords:** Family medicine concepts, Undergraduate curriculum, Communication, Patient, Centeredness, Empathy

## Abstract

**Background:**

The practice of family medicine is not well established in many developing countries including Sri Lanka. The Sri Lankan Government funds and runs the health facilities which cater to the health needs of a majority of the population. Services of a first contact doctor delivered by full time, vocationally trained, Family Physicians is generally overshadowed by outpatient departments of the government hospitals and after hours private practice by the government sector doctors and specialists. This process has changed the concept of the provision of comprehensive primary and continuing care for entire families, which in an ideal situation, should addresses psychosocial problems as well and deliver coordinated health care services in a society. Therefore there is a compelling need to teach Family Medicine concepts to undergraduates in all medical faculties.

**Discussion:**

A similar situation prevails in many countries in the region. Faculty of Medicine Peradeniya embarked on teaching family medicine concepts even before a department of Family Medicine was established. The faculty has recognized CanMed Family Medicine concepts as the guiding principles where being an expert, communicator, collaborator, advocate, manager and professional is considered as core competencies of a doctor. These concepts created the basis to evaluate the existing family medicine curriculum , and the adequacy of teaching knowledge and skills, related to family medicine has been confirmed. However inadequacies of teaching related to communication, collaboration, management, advocacy and professionalism were recognized. Importance of inculcating patient centred attitudes and empathy in patient care was highlighted. Adopting evaluation tools like Patient Practitioner Orientation Scale and Jefferson’s Scale of Empathy was established. Consensus has been developed among all the departments to improve their teaching programmes in order to establish a system of teaching family medicine concepts among students which would lead them to be good Family Physicians in the future.

**Summary:**

Teaching Family Medicine concepts could be initiated even before establishing departments of family medicine in medical faculties and establishing the practice of family medicine in society. Family medicine competencies could be inculcated among graduates while promoting the establishment of the proper practice of Family Medicine in the society.

## Background

The practice of family medicine is not well established in many developing countries. Sri Lanka is not an exception. The government delivers the major share of the health care free of charge. Doctors in the outpatient departments of major hospitals and all the doctors in smaller hospitals perform duties of a first contact doctor. Doctors working in the government sector have the privilege of engaging in private practice after working hours. This helps to generate additional income for them. As there is no established practice of family physicians in the country, doctors including specialists function as first contact doctors during private practice. In this back ground the Faculty of Medicine Peradeniya, the second largest medical faculty of the country, has recognized the responsibility of teaching attributes of a first contact doctor or family medicine concepts to all the undergraduates irrespective of their intentions on future specialization. However this teaching process is hampered not only by the lack of established system of family medicine in the country but also by the lack of a department of family medicine in this faculty. Teaching Family Medicine concepts deals with competencies rather than teaching knowledge and skills. All the departments recognized this requirement positively and feasibility of teaching family medicine concepts without a department of family medicine has been explored. This approach seems a better option in the current context of delivery of health care in the country.

### Main text

The background situation of the country relevant to practice and teaching Family Medicine was evaluated based on available documents. Current practice of teaching and possible areas of enhancing teaching was evaluated by a questionnaire survey and several focus group discussions conducted across all the departments in the faculty.

### Why family medicine should be taught

Family Medicine is defined as a specialty of medicine that provides comprehensive care to individuals and families by integrating biomedical, behavioral and social sciences [1-3]. Family physicians as first contact doctors provide primary and continuing care for entire families while addressing physical, psychological and social problems; and coordinates with specialists and other comprehensive health care services for the benefits of patients [3]. They practice patient centred medicine as oppose to doctor centred medicine. In patient centred care a patient is considered to be a unique individual and take his/her perspective into consideration in management of illness [4]. The exact definition of Family Medicine is constantly evolving and changing [3]. What is the best definition for a particular region or a country needs to be determined by the needs of that region or the country [5].

### Current situation in Sri Lanka

Sri Lanka is an island 65,610 km^2^ in size and a population of 21 million. In spite of relatively low annual per-capita gross national income of 5,520 USD the country enjoys a literacy ratio of 90%, average life expectancy of 75 years, infant mortality rate of 10.5 per 1000 live births and maternal mortality of 33.5 per 100,000 deliveries. Availability of physicians (4.9 per 10 000 population) is lower than the average in the region (5.50 per 10 000 thousand). However availability of nurses and midwives are higher (19.3 per 10,000 populations) than the average reported in the region (9.9 per 10 000 population). Soon after recovery from the 2004 tsunami and as an aftermath, the country is faced with the immediate challenges of a post war rehabilitation process. The emerging threats of increasing communicable diseases and non-communicable diseases in the near future has been recognized as even a bigger challenge for the health care systems of the country emphasizing the need for comprehensive primary health care service [6]. In addition first contact doctors are expected to manage a wide spectrum of problems expanding from acute infection related emergencies like sepsis, gastroenteritis, dengue, respiratory tract infections, emergencies related to non communicable disease, trauma and even medico legal problems.

Out of 15,977 doctors registered in the Sri Lanka Medical council in 2007, only 12% were in the full time private practice [7]. Out of the 1000–1500 doctors incorporated in to the doctor’s workforce annually, only a few will become specialists [8]. The Post Graduate Institute of Medicine in Sri Lanka conducts over 50 postgraduate courses and Family Medicine is one of them. Out of 2380 specialists trained by the PGIM from 1980 up to date only 18 had been on Family Medicine [8].

Majority of doctors will work as first contact doctors in their official duty or during their private practice during off-duty hours. Majority of the specialists also function as first contact doctors in the private sector. Therefore training family medicine concepts is relevant and imperative for all the undergraduates in Sri Lanka. However current practice of part time first contact private practice hinder establishment of a system of full time Family Medicine Doctors in the country. Some of the Medical Faculties have established their own Family Medicine Units for the purpose of teaching medical students. However there is no evidence of integration and expansion of the family medicine system in the country.

### The need has been recognized

The Scientific Working Group Meeting for the Development of a Core Curriculum in Family Medicine for the South-East Asian Region held in Colombo, Sri Lanka, in 2003, has recognized Family Medicine as a separate specialty and recommended to incorporate this subject into the existing medical curriculum and to establish Departments of Family Medicine with provision for the training of teachers in family medicine. The report emphasizes the need for post-graduate specialization and appropriate structures and cadres to incorporate trained family physicians within the national health system. Further this committee recommends the WHO support and collaborations with member countries in the region in order to achieve this target [9].

### Opinion of the faculty staff

Usage of an established competency framework like CanMED facilitated a rapid evaluation of the existing curriculum with regards to adequacy of teaching at department level. All the departments reported that the current practice of teaching knowledge and skills required for a first contact doctor as ‘adequate’. However teaching competencies in communication, collaboration, professionalism and management were considered ‘inadequate’. Everybody endorsed the need to teach these competencies with a view to producing a good quality doctor for the society.

## Discussion

### What is the emphasis in teaching?

Teaching Family Medicine demands teaching competencies expected by the society that extend beyond teaching facts and skills. The World Health Organisation (WHO) recognizes the importance of family medicine/primary care in its 2008 report; ‘primary care brings promotion and prevention, cure and care together in a safe, effective and socially productive way at the interface between the population and the health system’. WHO emphasizes patient centred care by "putting people first since good care is about people" [1,10]. As family medicine is based on patient centred care, patient’s expectations become very important. According to a customer survey conducted by St Vincent’s Hospital Melbourne, the most important attributes of a good doctor in order of significance includes; "caring, responsibility, empathy, interest, concern, competence, knowledge, confidence, sensitivity, perceptiveness, diligence, availability and manual skills" [1]. Dr Charles Boelen at the WHO (1993) defined family physicians/GP’s as "five star doctors" who can fulfil five essential functions; "assess and improve quality of care by responding to the patients total health needs, make optimal use of new technologies, promote healthy life-style, record individual and community health requirements and work efficiently in a team" [1]. Jhone Murtagh 2001 has proposed 10 guiding rules for a good GP; "developing rapport and good communication skills, asking the right question, be astute and observant, develop optimal ethical and professional standards, having a fail-safe diagnostic strategy, developing supportive network, knowing essential therapeutics, developing basic procedural skills, being well prepared for emergencies and knowing yourself and your limitations" [1]. Producing this ‘good doctor’ is a challenge for medical educators. The system of medical education in a country could be revised and modified to fulfil the need of that society [11]. Undergraduate and postgraduate courses should have reciprocal contributions, shared modules, for teaching undergraduates and training registrars [11,12].

Family Physicians are community based, skilled clinicians; serving a defined population, based on doctor patient relationships (CanMED competencies) The doctor patient relationship expects the competencies as a communicator, collaborator, and a professional. Skilled clinician refers to being an expert,, a communicator and a scholar. Skills in collaboration, management and advocacy become essential for a community based practice. As family physicians are resources to a defined population their competencies as a collaborator, manager, advocate and a scholar become valuable. Theses competencies will add on to the qualities of a good doctor [13].

### Teaching communication skills

Communication is an essential competency of a doctor that should be effectively integrated with other competencies related to knowledge, skills, and problem-solving to become a good quality doctor [14]. Doctors need to communicate with patients, co-workers, administrators, and the public. Poor communication results in failure to recognize patients presenting complaint [15] their concerns [16] and their needs with regards to management of their medical problems. Health professionals should unite the artistic and humanistic side of care with the technical side and learn how to be a professional without losing their humanistic identity [17-19] while perceiving and learning from patients’ narratives [20].

Communications with patients ensure patient satisfaction and doctor patient relationship [21], facilitates discovering the entire health problem [22], and helps to understand and establish the diagnosis. Good communication helps in management of patients, achieving better therapeutic adherence [23] and it can be therapeutic [24,25] and cost effective [26] Doctors who communicate well with patients are less likely to be sued, and suffer vicarious trauma [26].

History taking is the most commonly practiced communication skill by students and doctors. The current practice of teaching emphasizes information gathering marginalizing its psychosocial, professional and artistic aspects. Patient centred history-taking emphasis asking open-ended questions and effective listening to accommodate total stories and perspectives of patients. This practice could be established by teaching training as well as by introducing a structured feedback from peer students and patients. A feedback form developed by the Calgary Cambridge University is a good example to adopt [17]. Empathy is the precursor of attitudes; altruism, communication, patient centeredness and ultimately overall performance of a doctor depend on empathy that can be inculcated among students by teaching and training [27].

### Assessment of attitude changes

Teaching without an assessment is likely to fail. Assessment of attitudes is much more difficult than evaluation of knowledge and skills. Numerous self-reporting and observer based tools have been developed [28]. Patient Practitioner Orientation Scale (PPOS) is a simple, short and reliable self-reporting tool for monitoring and research to evaluate patient centeredness among doctors, patients and students [29]. Jefferson’s Scale of Empathy assesses empathy of students and doctors [30] and it has helped to recognize deterioration of empathy during clinical practice [30]. Developing methods to evaluate patient’s perception of physician’s empathy would be a valuable intervention to inculcate patient centred attitudes among students [31].

However using tools developed in the Western World to assess attitudes in a different cultural background is a debatable topic. One may argue that values expressed by those tools are not valid for comparisons across cultures. But this does not preclude using these tools for comparison within the same culture or to recognize trends in a cohort. Inner needs of human have much more similarities than subtle differences influenced by the culture. Evaluation of attitudes and empathy in groups of people will create awareness and initiate a dialogue in a society. That will be conducive for further refining of these tools or development of new tools and research to address the need of the specific society.

### Collaborative, managerial and advocacy skills

Medicine cannot be practiced in isolation. It involves multiple agencies and professionals. Collaborative skills are essential. Our graduates are appointed as administrators of institutions very early in their careers that require skills to manage institutions and human resource. They have to prioritize and use health resources wisely, and effectively to execute tasks collaboratively with colleagues. Implementing policies like rational use of drugs, evidence based medicine and clinical audits become important skills for primary care doctors. Issues related to patient satisfaction, patient waiting time and cost of care come under their purview. It is important to sensitize undergraduate regarding this aspect of overall duties that they have to look after as doctors in the future. Doctors in the preventive sector have an official role as health advocates. Even without such positions doctors in our society become advocates of health care policies automatically. Their role as advocates extends beyond individual patients and families. They get involved in schools, temples and the entire society enabling them to make a major contribution in health promotion and integrating health care with society. These aspects of advocacy skills could be sharpened during the undergraduate curriculum so that they become effective and useful to the society.

### Skills as a scholar and a researcher in the society

A doctor needs to be a scholar and demonstrate lifelong commitment to reflective learning. They should contribute to dissemination and application of new knowledge. Their contribution to research and teaching students will be invaluable to society. Their observations can be very valuable for progress in science as they handle undifferentiated patients directly in the society. They have the best opportunities to make observations in the society. Undergraduate curriculum should create the basis for doctors to function as scholars while they practice, irrespective of their positions.

### Professionalism

Professionalism encompasses set of attitudes; values and behaviours expected by the society and other stakeholders [32]. Professionalism in the undergraduate curriculum is limited to teaching practices like obtaining consent, having a chaperone in clinical examination etc. At present behavioural changes are not actively taught but expected to permeate to students by passive role modelling of teachers [33]. Teachers notion that that attitudes cannot be taught needs to be changed by incorporating discussion on methods of teaching professionalism and attitudes in the faculty development program [33].

### Barriers to establish a system of family physicians

Possibility of considering family medicine as a separate subject/discipline or is it the practice of what is learnt in other major disciplines of medicine is being debated. An academic discipline should have its defined content area, teaching methodologies, comprehensive established health care services, aligned with undergraduate and postgraduate education and research [34]. Family medicine conforms to all these requirements in many countries. However, introducing a Department of family medicine to Medical Faculty in a country without an established family practice can be the subject for controversy [34]. To establish a system of Family Physicians in a country the field of family medicine should attract doctors. At present Family Medicine is not a popular career choice among doctors in Sri Lanka, which is reflected by the minority who have selected Family Medicine as their specialty. Attraction to become a specialist due to its capacity to earn higher income and social recognition has superseded the desire to become Family Physicians. It is rare for a doctor to decide to take up Family Medicine as their first carrier choice at the onset. Professional identity, ill-defined area of expertise, conflict about their role and lack of academic culture in the discipline of family medicine are some of the reasons that make it less attractive [35]. Teachers who are specialists do not appear as attractive role models as generalists and they tend to emphasise their work as specialists. Mistakes and deficiencies of the first contact doctors becomes the teaching point in ward classes. Students perceive Family Medicine as a less important specialty. Establishing family practice in a country needs dedicated evaluation of the current practices and needs of the society [12,36].

## Conclusion

Teaching family medicine concepts to all the graduates is an imperative need of society. Majority of doctors function as fist contact doctors even after specializing in their careers. Therefore all the doctors should acquire attributes of a family physician. This process should be initiated during the undergraduate training. Therefore undergraduate curriculum needs to be revisited with the intention of enhancing the teaching Family Medicine competencies even before establishing departments of Family Medicine or a system of Family Medicine in the society. This process will establish teaching competencies rather than facts and skills in undergraduate curriculum and would the basis to establish a system of Family Medicine in a country.

## Abbreviations

PPOS: JSE.

## Competing interest

The author declare that he has no competing interest.

## Authors’ contribution

RMM did literature survey conducted opinion survey and focus group discussion and finally prepared the manuscript. Interventions are in progress.

## Authors’ information

Dr R.M. Mudiyanse is the Head of the Department of Pediatrics in the Faculty of Medicine, Peradeniya, Sri Lanka. He has worked as a first contact doctor in the Ministry of Health Sri Lanka for 12 years before joining the University. Faculty of Medicine Peradeniya has entrusted him to formulate measures to enhance teaching family medicine concepts in the faculty curriculum even before establishing a separate department of family medicine. He has been selected for FAIMER fellowship program to conduct this project.
